# Clinical Outcomes of Transverse Mini-Port Incision Versus Conventional Carpal Tunnel Release: A Randomized Comparative Analysis of Patients With Carpal Tunnel Syndrome

**DOI:** 10.7759/cureus.92190

**Published:** 2025-09-13

**Authors:** Vivek Gupta, Sudipta Bera, Nikhil Das, Sandip Basu

**Affiliations:** 1 Department of General Surgery, G.S. Medical College and Hospital, Hapur, IND; 2 Department of Plastic and Reconstructive Surgery, Institute of Postgraduate Medical Education and Research and Seth Sukhlal Karnani Memorial Hospital (IPGMER and SSKM Hospital), Kolkata, IND; 3 Department of Plastic and Reconstructive Surgery, Banaras Hindu University and Institute of Medical Sciences, Varanasi, IND; 4 Department of General Surgery, PES Institute of Medical Sciences and Research (PESIMSR), Kuppam, IND; 5 Department of Plastic and Reconstructive Surgery, R.G. Kar Medical College and Hospital, Kolkata, IND

**Keywords:** carpal tunnel release, mini incision, port incision, transverse incision, wrist crease incision

## Abstract

Background: Carpal tunnel release (CTR) is conventionally performed with a longitudinal mid-palmar incision, but it is reported to have incision-related complications. Various modifications, including endoscopic CTR and several mini-incision methods, have been described to reduce these complications. However, the literature on a transverse wrist crease incision is rare. Here, we describe a CTR method using a transverse mini-port incision, initially employed as a port for endoscopic CTR. This randomized controlled study aims to evaluate the safety and effectiveness of the transverse mini-port incision CTR and compare its outcomes with those of the conventional open method.

Materials and methods: The study includes 60 upper limbs operated with either the conventional open method (Group A, n = 30) or the transverse mini-port incision method (Group B, n = 30) at a tertiary teaching hospital between January 2015 and December 2016. The patients were assessed and compared using the Levine Score, Scar Tenderness VAS Score, and Pillar Pain VAS Score on the seventh day, at six weeks, at 26 weeks, and at 36 weeks, along with return to activities postoperatively.

Results: The return to daily activities was earlier in Group B. There was significantly less scar tenderness in Group B on the seventh day and six weeks postoperatively; however, long-term follow-up showed no significant difference between the two groups.

Conclusions: The transverse mini-port incision is a safe and beneficial alternative to conventional open CTR. For patients who are concerned about aesthetic results and early return to work, it may be a simple, safe, and inexpensive alternative to other mini-incision methods.

## Introduction

Carpal tunnel syndrome (CTS) is the most common compression neuropathy of the upper limb. CTR is conventionally performed with a longitudinal incision along the median palmar crease, allowing for direct visualization of the anatomical structures and effective decompression. The conventional open method is presently regarded as the gold standard [1]. However, it is associated with incision-related complications such as hypertrophic scar, scar tenderness, and prolonged pillar pain [2]. To reduce these complications, various modifications have been introduced, including endoscopic CTR and several mini-incision methods.

Most of these mini-incision methods use shorter longitudinal mid-palmar incisions and are reported to improve scar-related complications, pillar pain, and functional recovery [3,4]. However, the availability of endoscopes and dedicated instruments remains a concern. Moreover, neurovascular injury, incomplete release, and other local complications have been attributed to the limited exposure. Although numerous reports comparing mini-incision methods have been published, reports on transverse incision methods are rare.

Here, we describe a CTR method with a transverse mini-port incision at the wrist crease, initially used as a port for endoscopic CTR. No dedicated instruments other than standard hand surgery instruments were required. This prospective study aims to evaluate the safety and effectiveness of the transverse mini-port incision CTR and compare its outcomes with the conventional open method.

## Materials and methods

This prospective comparative interventional study was conducted at the Institute of Postgraduate Medical Education and Research and Seth Sukhlal Karnani Memorial Hospital in Kolkata between January 2015 and December 2016. The study was approved by the Institutional Ethics Board of the Institute of Postgraduate Medical Education and Research (approval number: Inst/IEC/2015/172) and adhered to the ethical standards outlined in the 1964 Declaration of Helsinki and its subsequent amendments.

All patients attending the Plastic Surgery Outpatient Department with complaints suggestive of CTS were evaluated clinically and with electromyography and nerve conduction studies. Patients were first given a trial of conservative treatment with splinting and steroid injection. Those who did not benefit from conservative management for more than one month were selected for surgical treatment and included in the study. Patients with diabetes mellitus, chronic renal failure, hypothyroidism, psychosis, or a history of previous CTS surgery were excluded. Informed written consent was obtained for the surgery, participation in the study, and anonymized data utilization from each participant. Patients who declined consent were excluded. Standard guidelines were followed throughout the study and in the reporting of this article.

A total of 60 upper limbs from 60 patients with CTS were recruited and randomized to undergo either the conventional open method (Group A) or the transverse mini-port incision method (Group B). Each group consisted of 30 patients, with a 95% confidence level, 80% power, and an effect size of 0.8. Random allocation was performed using a computer-generated random number table.

Surgical methods

All procedures were performed under local anesthesia as office-based surgeries by the same surgeon. The wrist was positioned in extension over a rolled towel. CTR was performed using either the conventional open method or the transverse mini-port incision method.

Conventional Open CTR

In the conventional open group, a longitudinal incision was made between the thenar and hypothenar prominences, starting from the distal wrist crease and extending approximately 2.5-3 cm distally over the carpal tunnel. The distal limit corresponded to Kaplan’s cardinal line. The transverse carpal ligament (TCL) was exposed with blunt dissection and divided to the distal limit under direct vision.

Transverse Mini-Port Incision CTR

**Figure 1:**
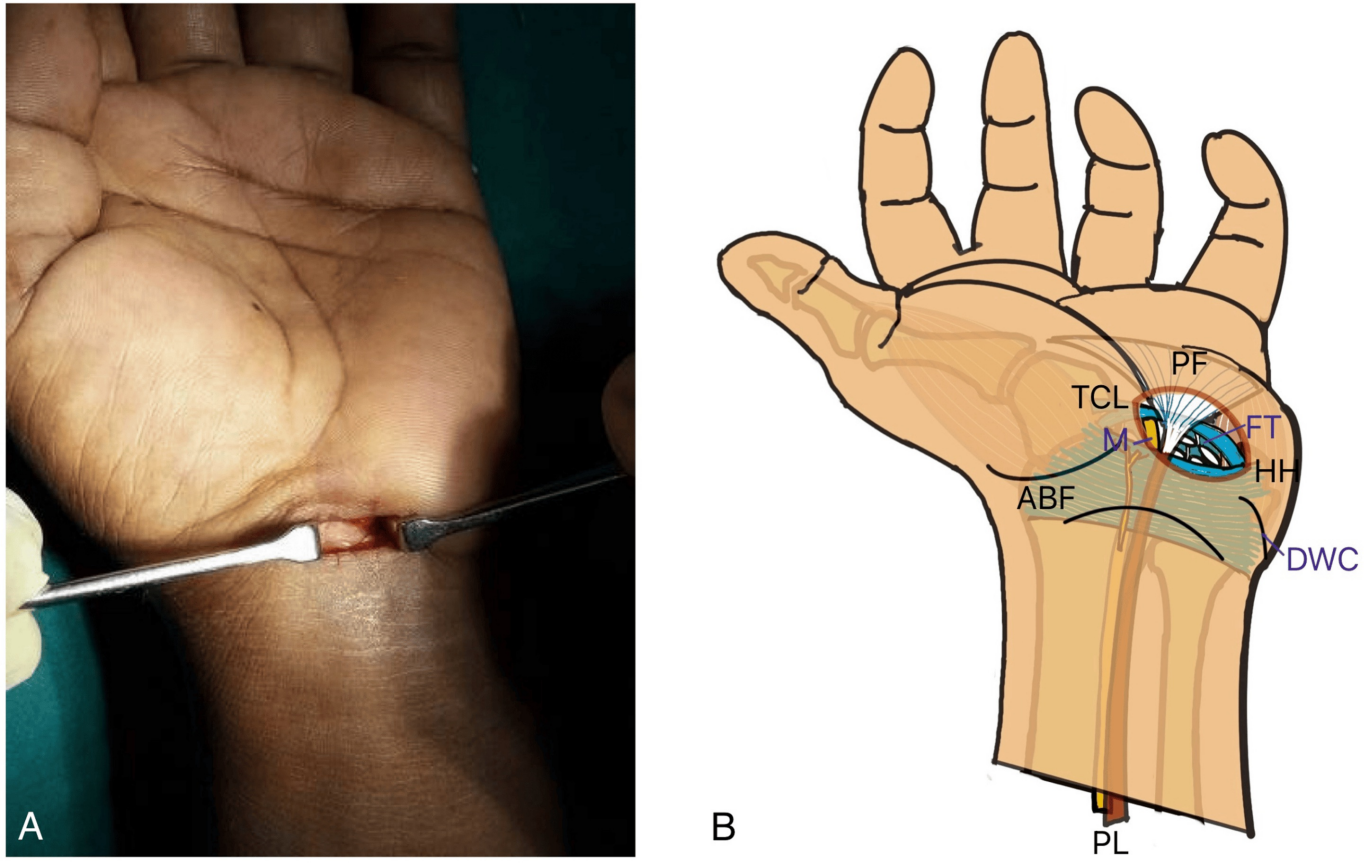
Transverse mini-port incision method (A) Clinical intraoperative picture of the mini-port incision. (B) Diagrammatic illustration showing the anatomical landmarks. PF: palmar fascia, ABF: antebrachial fascia, TCL: transverse carpal ligament, M: median nerve, FT: flexor tendon, HH: hook of hamate, DWC: distal wrist crease, PL: palmaris tendon Image Credit (B): Authors

In the transverse mini-port incision group, an approximately 1.5 cm incision was made on the distal wrist crease between the long axis of the fourth webspace and the palmaris longus (PL) tendon. With the wrist extended, the roof of the carpal tunnel remained at the apex of the palm and wrist, making it easily accessible through the wrist crease incision (Figure 1). The PL tendon was identified and retracted radially with a cat’s paw retractor to expose the antebrachial fascia. At the roof of the carpal tunnel, the PL tendon blends with the palmar fascia superficially, and the antebrachial fascia blends with the proximal margin of the TCL deeply. The loose plane between these fascial layers was opened with blunt dissection using a peanut gauze. A 5 mm transverse incision was made with a scalpel on the antebrachial fascia to open the tunnel. Closed scissors were advanced proximally to create an extrabursal path under the antebrachial fascia, and the distal portion was divided with blunt scissors to reach the proximal margin of the TCL. Closed scissors were then advanced distally to establish an extrabursal path under the ligament, allowing visualization of the tunnel. Anatomical variations of the median nerve were addressed, and the proximal margin of the TCL was divided under direct vision. Retraction of the distal margin of the incision with the palmar fascia improved visibility, and the distal portion of the ligament was divided with blunt straight scissors. Transection was advanced snip by snip along the longitudinal axis of the fourth digit until a characteristic “snap” indicated completion.

After the TCL division in both groups, completeness of release was assessed. Closed straight scissors or the back of a forceps was passed distally beyond the release and slowly withdrawn while directed volarly; a palpable step indicated incomplete release. The median nerve was also visualized to confirm effective decompression. The skin incision was closed with 3-0 non-absorbable monofilament sutures.

Gentle, active finger movements and hand elevation were encouraged immediately postoperatively. Analgesics were prescribed for the first 48 hours and subsequently as needed. Patients were followed up after one week, and sutures were removed between days 7 and 10.

Follow-up

Patients were assessed at day 7 and subsequently at 6, 12, and 26 weeks postoperatively. Symptom severity and functional outcomes were evaluated using the Levine Score, which comprises the Symptom Severity Scale (SSS) and the Functional Status Scale (FSS). The SSS includes 11 self-administered items related to pain, numbness, tingling, and weakness, while the FSS includes eight items concerning functional difficulties such as writing, buttoning clothes, gripping objects, opening jars, household chores, carrying groceries, and self-care activities. Each item was scored from 1 to 5, with 5 indicating the most severe impairment [5]. Postoperative scores were compared with preoperative scores to objectively assess symptom resolution and functional improvement. Scar tenderness and pillar pain (tenderness at the thenar and hypothenar bases) were evaluated using a visual analog scale (VAS). Patients also recorded the time taken to return to daily activities (in days). All patients were followed for at least one year, with additional follow-ups as needed.

Statistical analysis

Continuous variables were expressed as mean ± standard deviation (SD), and categorical variables as counts and percentages. Student’s independent t-test was used to compare continuous variables, while the chi-square test or Fisher’s exact test was used for categorical variables. Statistical significance was set at p ≤ 0.05, with results expressed as p-values and chi-square (χ²) values.

## Results

In our study, 30 patients were included in each group for comparison. There were no significant differences between the groups with respect to age, sex, side of involvement, laterality, and preoperative Levine Score (Table 1).

**Table 1:** Comparison of baseline characteristics between the groups SD: standard deviation, n: number, FSS: Functional Status Scale, SSS: Symptom Severity Scale The original source has been cited for the Levine Score and the FSS and SSS scales used in the study. Proof of permission was obtained from the copyright holder [5].

Characteristics	Group A (n = 30)	Group B (n = 30)	p-value/ꭓ2value
Age mean (SD)	41.13 (7.71)	43.33 (8.36)	p = 0.29
Sex (n)	Male (3); female (27)	Male (4); female (26)	p = 0.69, ꭓ2 = 0.16
Side (n)	Left (12); right (18)	Left (9); right (21)	p = 0.42, ꭓ2 = 0.66
Hand involvement (n)	Unilateral (7); bilateral (23)	Unilateral (8); bilateral (22)	p = 0.94, ꭓ2 = 0.13
Preoperative FSS mean (SD)	2.61 (0.31)	2.60 (0.29)	p = 0.96
Preoperative SSS mean (SD)	2.80 (0.24)	2.75 (0.24)	p = 0.49

**Figure 2:**
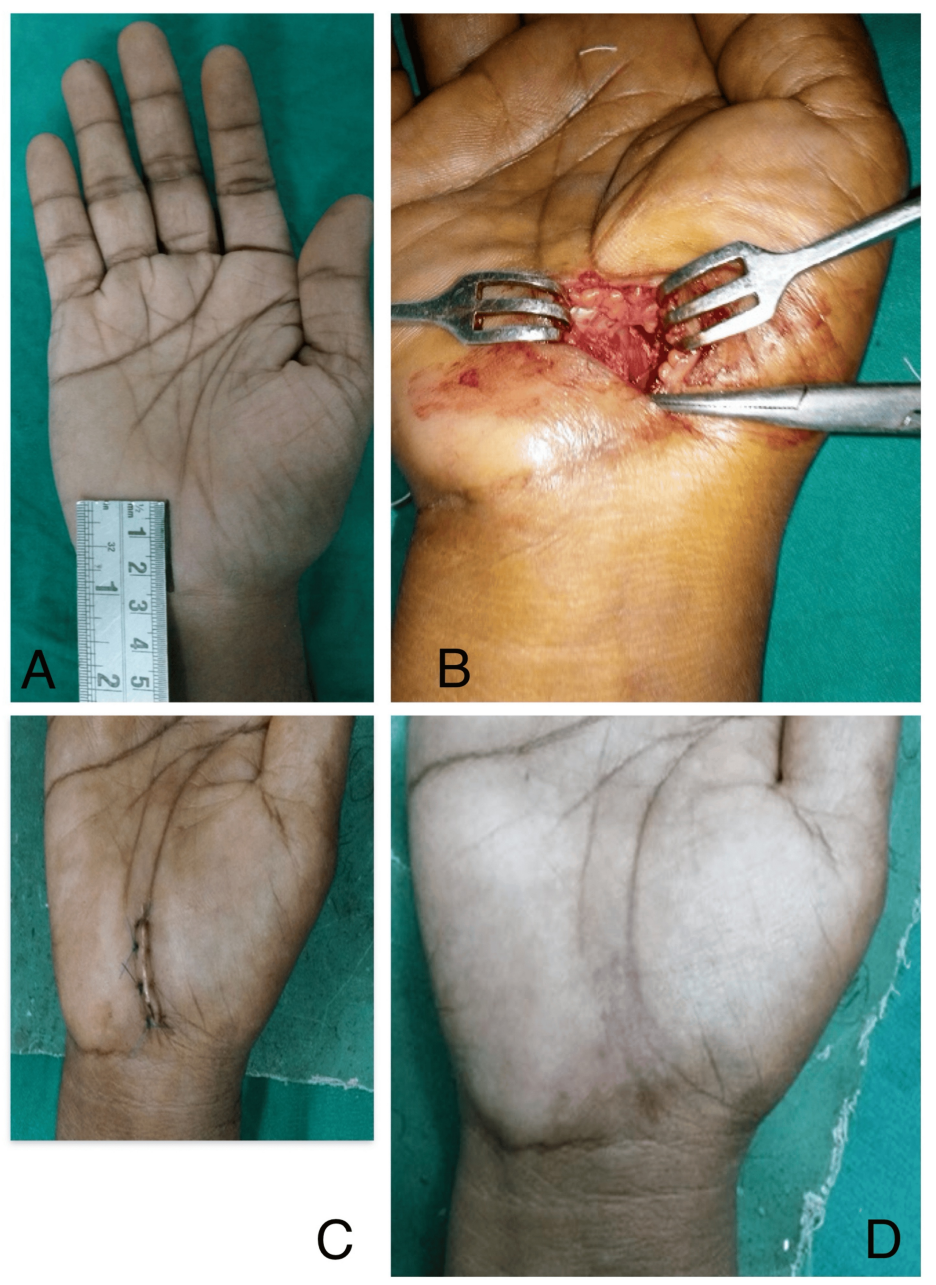
Clinical photographs of CTR with conventional incision (Group A) (A) Incision marking. (B) Intraoperative photo showing the extent of dissection. (C) Seventh-day postoperative follow-up. (D) 26th-week postoperative follow-up. CTR: carpal tunnel release

**Figure 3:**
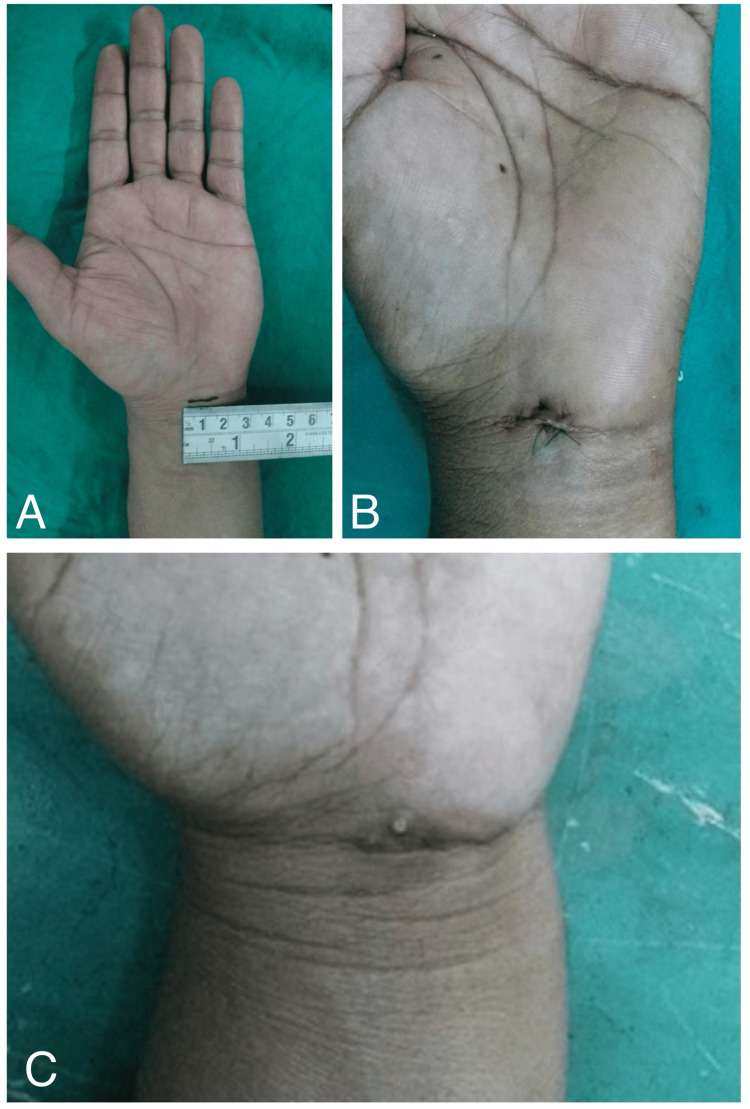
Clinical photographs of CTR with transverse mini-port incision (Group B) (A) Marking of incision. (B) Seventh-day postoperative follow-up. (C) 26-week postoperative follow-up. CTR: carpal tunnel release

We observed that CTS is most common in individuals aged 40-60 years. It was more prevalent in females (88.3%) than in males (11.7%), with a sex ratio of 7.57. Bilateral involvement was seen in 75% of patients, whereas 25% had unilateral disease. Incision lines and scars in both groups are shown in Figures 2-3.

At the sixth postoperative week, patients in the transverse mini-port incision group had significantly lower functional severity scores compared with the conventional incision group (Table 2). At the 12th postoperative week, symptom severity scores were also significantly lower in the transverse mini-port incision group (Table 3).

**Table 2:** Follow-up FSS between the groups FSS: Functional Status Scale, SD: standard deviation The original source has been cited for the Levine Score and the FSS and SSS scales used in the study. Proof of permission was obtained from the copyright holder [5].

Follow-up period	Group A mean (SD)	Group B mean (SD)	p-value
7th day	1.98 (0.076)	1.99 (0.086)	0.84
6th week	1.33 (0.16)	1.17 (0.17)	0.0009
12th week	1 (0)	1 (0)	1
26th week	1 (0)	1 (0)	1

**Table 3:** Comparison of follow-up SSS between the groups SSS: Symptom Severity Scale, SD: standard deviation The original source has been cited for the Levine Score and the FSS and SSS scales used in the study. Proof of permission was obtained from the copyright holder [5].

Follow-up period	Group A mean (SD)	Group B mean (SD)	p-value
7th day	1.58 (0.14)	1.57 (0.12)	0.86
6th week	1.41 (0.091)	1.43 (0.088)	0.51
12th week	1.31 (0.045)	1.30 (0.043)	0.43
26th week	1.042 (0.061)	1.045 (0.052)	0.83

Scar tenderness was significantly lower in the transverse mini-port incision group at the first and sixth postoperative weeks. However, there was no difference between groups in terms of pillar pain (Tables 4-5).

**Table 4:** Comparison of scar tenderness between the groups VAS: visual analog scale, n: number of patients

Follow-up period	VAS score	Group A (n)	Group B (n)	p-value/ꭓ2 value
7th day	2	0	10	p ≤ 0.0001, ꭓ2 = 43.00
	3	4	19	
	4	17	1	
	5	9	0	
6th week	1	4	26	p ≤ 0.00001, ꭓ2 = 32.27
	2	20	3	
	3	6	1	
12th week	1	30	30	
26th week	1	30	30	

**Table 5:** Comparison of pillar pain between the groups VAS: visual analog scale, n: number of patients

Follow-up period	VAS score	Group A (n)	Group B (n)	p-value/ꭓ2 value
7th day	1	0	0	p = 0.076, ꭓ2 = 6.87
	2	5	7	
	3	13	17	
	4	12	4	
	5	0	2	
6th week	0	5	4	p = 0.77, ꭓ2 = 0.53
	1	23	25	
	2	2	1	
	3			
	4	0	2	
12th week	0	5	4	p = 0.77, ꭓ2 = 0.52
	1	23	25	
	2	2	1	
26th week	0	10	6	p = 0.24, ꭓ2 = 1.36
	1	20	24	

The mean return-to-work time was 22.26 (SD 3.95) days in the conventional incision group and 13.06 (SD 2.67) days in the transverse mini-port incision group, with the difference being statistically significant (p < 0.0001). Hypertrophic scar formation was observed in two patients in Group A.

## Discussion

Surgical methods for CTR have been modified over the years since its first description in 1930. CTR can be broadly divided into three methods: the open method, the endoscopic method, and the mini-open method. In the open method, the carpal tunnel is approached through a mid-palmar incision, whereas in the endoscopic method, it is approached through a wrist crease incision. In the mini-open method, the distal portion of the open method incision is used, although numerous variations have been reported [6].

Conventional open method

**Figure 4:**
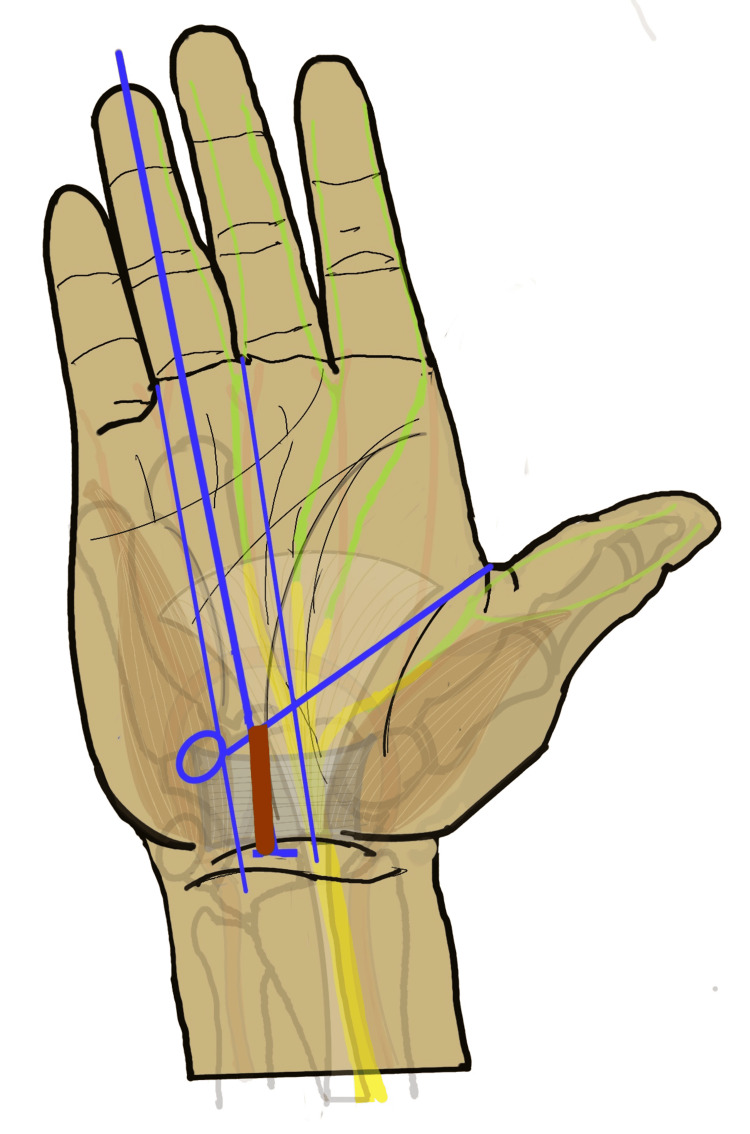
Incision for conventional open CTR CTR: carpal tunnel release Incision line marked with brown. Image Credit: Authors

The conventional open method involves a 4-5 cm longitudinal incision between Kaplan’s cardinal line and the proximal wrist crease (Figure 4).

This approach allows direct visualization of the ligament and tunnel contents, ensuring a complete division of the TCL and identification of anatomic variations or associated pathologies. It also reduces the risk of injuring an intraligamentous palmar cutaneous branch of the median nerve through direct dissection [7]. However, the drawbacks include scar tenderness, pillar pain, delayed healing, bowstringing of the flexor tendons, reflex sympathetic dystrophy, tendon adhesion, and cosmetic concerns [8]. These complications are thought to arise from injury to the dermal sensory plexus and distal branches of the palmar cutaneous branch of the median nerve during incision. Longer incisions increase the risk of these issues [9]. Currently, many surgeons prefer shorter incisions extending between Kaplan’s cardinal line and a point 1-2 cm distal to the distal wrist crease, which provides sufficient exposure [10].

Endoscopic method

Introduced in 1989, the endoscopic method aimed to reduce incision-related complications. With this approach, the carpal tunnel is accessed through a small incision, leaving the palmar skin intact [11]. The TCL is divided into endoscopic visualization, either through the same incision or a separate port. Reported advantages include less scar tenderness, reduced pillar pain, earlier return to daily activities, and improved cosmetic satisfaction [12]. The wrist crease is the typical site for the port incision, although the mid-palmar crease can also be used [13,14].

Despite these benefits, the endoscopic method is limited by the need for specialized equipment and surgical expertise. Reported drawbacks include higher risks of neurovascular injury, incomplete release, and symptom recurrence due to restricted exposure. Consequently, the cost-effectiveness of this technique remains uncertain [15].

Mini-open method 

**Figure 5:**
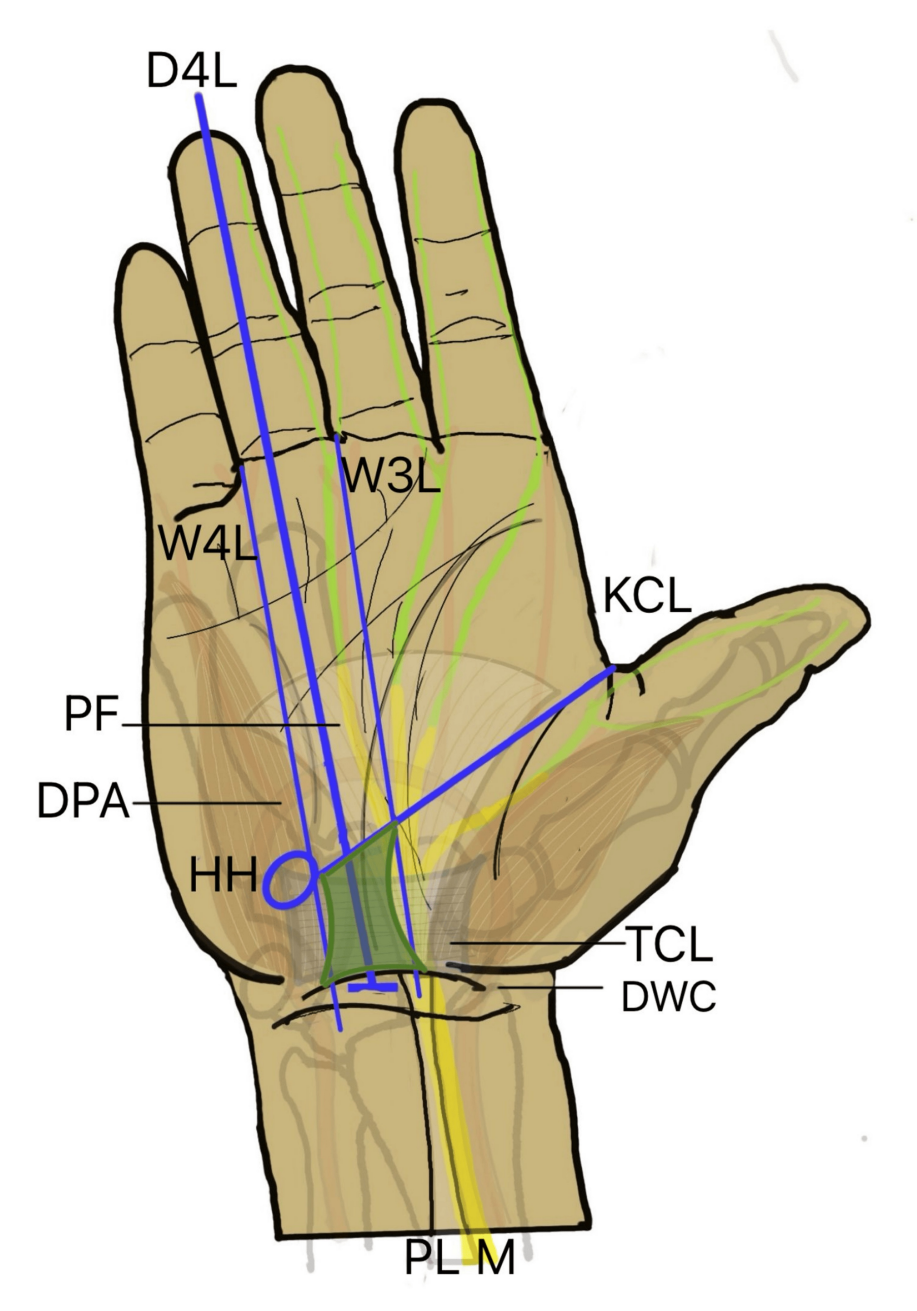
Anatomical landmarks for safe exposure of the carpal tunnel The safe zone for CTR is marked as an hourglass-shaped green area between the thenar and hypothenar muscles. D4L: long axis of fourth digit, 3WL: long axis of the third web space, 4WL: long axis of the fourth web space, KCL: Kaplan’s cardinal line, PF: palmar fascia, DPA: deep palmar arch, HH: hook of hamate, TCL: transeverse carpal ligament, DWC: distal wrist crease, PL: palmaris longus tendon, M: median nerve, CTR: carpal tunnel release Image Credit: Authors

To combine the exposure advantages of the open method with the reduced scar-related complications of the endoscopic method, various mini-open techniques have been developed. These limited-incision approaches rely on anatomical landmarks to reduce the risk of neurovascular injury (Figure 5) [3,6].

**Figure 6:**
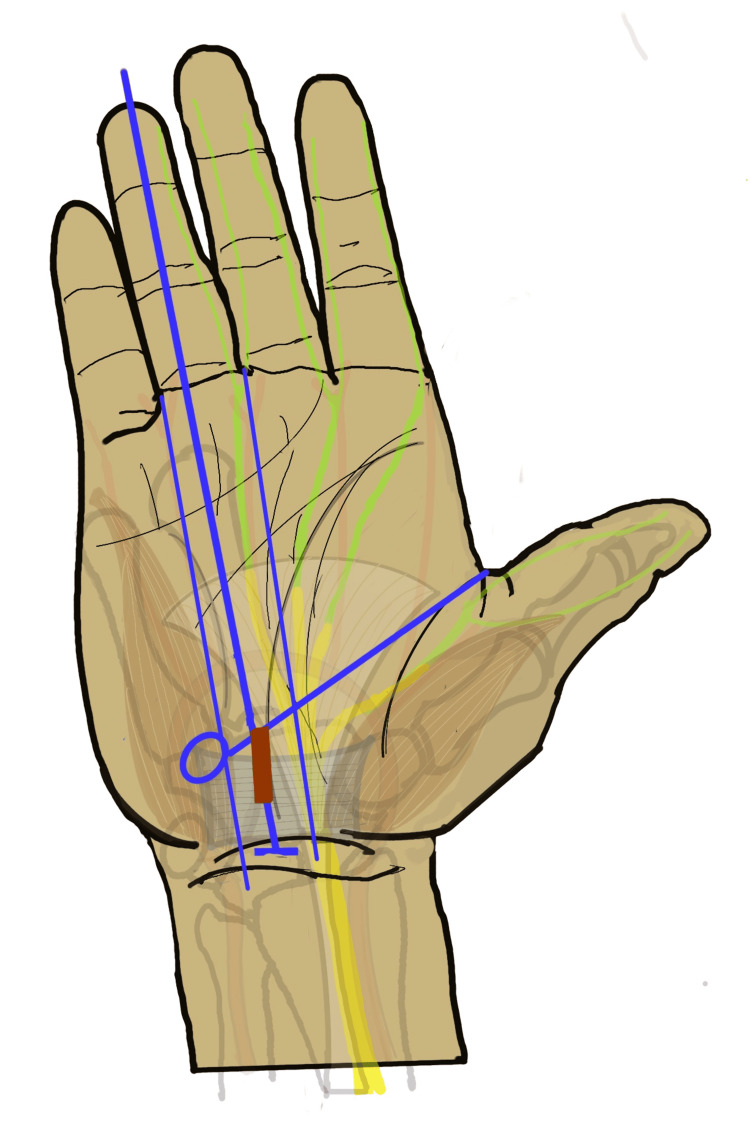
Usual incision line for mini-open CTR CTR: carpal tunnel release The incision line is marked with brown. Image Credit: Authors

Most mini-open methods target the distal-most limit of the TCL, near the vascular arch. Kaplan’s cardinal line, line-approximately 2.5 cm proximal to the deep palmar arch and 1 cm distal to the distal TCL margin, serves as a guide to the distal limit. A shorter incision is then placed along the mid-palmar crease, usually between the long axis of the third and fourth webspaces (Figure 6).

The TCL is generally approached distally to proximally, though variations exist in incision size, number, and placement [16]. Specialized retractors may be used to enhance visibility in cases of limited exposure [17-19]. Mini-open methods are associated with reduced scar tenderness, pillar pain, and earlier recovery, though incomplete release and proximal nerve injury remain concerns [20,21].

Transverse mini-port incision method

Several studies have examined mini-incision techniques and compared them with conventional or endoscopic approaches [22,23]. However, descriptions of the transverse mini-port incision method are rare [6,14]. This study aimed to achieve the benefits of a mini-incision procedure while maintaining cost-effectiveness by using standard hand surgery instruments.

The transverse mini-port incision produced better cosmetic outcomes, as the incision was aligned with the distal wrist crease and required less deep dissection. The procedure reduced pillar pain and scar tenderness, with results comparable to those of endoscopic and other mini-incision methods [24,25].

In mini-open approaches with longitudinal incisions, proximal exposure of the carpal tunnel raises the risk of injury to the palmar cutaneous branch, muscular branches, and the median nerve [26,27]. By contrast, the transverse mini-port incision at the distal wrist crease allows the proximal part of the tunnel to be approached under direct vision. This horizontal incision provides better visualization of the proximal TCL margin compared with a longitudinal incision. Since the TCL is divided proximally to distally, the distal end is cut blindly. However, using Kaplan’s cardinal line and the proximal TCL margin as landmarks, similar to the conventional open method, helps minimize the risk of vascular or nerve injury. In our study, no injuries to the median nerve or flexor tendons occurred in either group.

Regardless of the method, the primary objective is complete decompression of the carpal tunnel. In our study, both surgical techniques achieved full release of the TCL and decompression of the median nerve. With standardized intraoperative methods to confirm completeness of release, no recurrences were observed during the follow-up period.

Limitations

The main limitation of this study is its small sample size. Larger, multi-institutional studies are needed to provide stronger evidence.

## Conclusions

For patients concerned about cosmetic outcomes and an early return to work, the transverse mini-port incision CTR offers a valuable alternative to conventional techniques. Our findings suggest that this approach provides more comfortable short-term postoperative outcomes, promotes faster functional recovery, and minimizes iatrogenic injury. In addition, the scar remains concealed within the wrist crease, contributing to greater patient satisfaction. While the conventional method may still be preferable in cases of recurrence, the transverse mini-port incision appears to be a safe, simple, and cost-effective option with distinct cosmetic and functional advantages over the standard approach.
